# Intralimb Coordination Patterns in Absent, Mild, and Severe Stages of Diabetic Neuropathy: Looking Beyond Kinematic Analysis of Gait Cycle

**DOI:** 10.1371/journal.pone.0147300

**Published:** 2016-01-25

**Authors:** Liu Chiao Yi, Cristina D. Sartor, Francis Trombini Souza, Isabel C. N. Sacco

**Affiliations:** 1 Federal University of São Paulo, Santos, Brazil; 2 Physical Therapy, Speech and Occupational Therapy Department, University of Sᾶo Paulo, São Paulo, Brazil; 3 Department of Physical Therapy, University of Pernambuco, Petrolina, Brazil; Harvard Medical School, UNITED STATES

## Abstract

**Aim:**

Diabetes Mellitus progressively leads to impairments in stability and joint motion and might affect coordination patterns, mainly due to neuropathy. This study aims to describe changes in intralimb joint coordination in healthy individuals and patients with absent, mild and, severe stages of neuropathy.

**Methods:**

Forty-seven diabetic patients were classified into three groups of neuropathic severity by a fuzzy model: 18 without neuropathy (DIAB), 7 with mild neuropathy (MILD), and 22 with moderate to severe neuropathy (SVRE). Thirteen healthy subjects were included as controls (CTRL). Continuous relative phase (CRP) was calculated at each instant of the gait cycle for each pair of lower limb joints. Analysis of Variance compared each frame of the CRP time series and its standard deviation among groups (α = 5%).

**Results:**

For the ankle-hip CRP, the SVRE group presented increased variability at the propulsion phase and a distinct pattern at the propulsion and initial swing phases compared to the DIAB and CTRL groups. For the ankle-knee CRP, the 3 diabetic groups presented more anti-phase ratios than the CTRL group at the midstance, propulsion, and terminal swing phases, with decreased variability at the early stance phase. For the knee-hip CRP, the MILD group showed more in-phase ratio at the early stance and terminal swing phases and lower variability compared to all other groups. All diabetic groups were more in-phase at early the midstance phase (with lower variability) than the control group.

**Conclusion:**

The low variability and coordination differences of the MILD group showed that gait coordination might be altered not only when frank evidence of neuropathy is present, but also when neuropathy is still incipient. The ankle-knee CRP at the initial swing phase showed distinct patterns for groups from all degrees of neuropathic severity and CTRLs. The ankle-hip CRP pattern distinguished the SVRE patients from other diabetic groups, particularly in the transitional phase from stance to swing.

## Introduction

The study and description of alterations in the gait biomechanics of diabetic patients have been an issue for at least 40 years [1–7]. Because the most devastating comorbidities are related to gait deviations, such as foot ulceration [8, 9] and falls [10], understanding how those alterations are perceived has been believed to guide efficient therapeutics seeking to avoid the tragic consequences of diabetes progression.

Diabetes, especially associated with polyneuropathy, is known to cause several impairments in stability and mobility, and affects the harmonious interaction between muscles, joints, and sensory information during locomotor tasks. Literature on the diabetic patient gait biomechanical patterns has described the walk as slow, with wider steps, a reduction in ankle range of motion [4, 5], a shorter center of pressure excursion [11], higher operating ankle and knee strength [12], and higher peak pressure over the forefoot [6].

Frequently, the reduction in the range of motion of one distal joint, which is commonly observed in the diabetic population [5, 13, 14], can lead the adjacent joints to adapt to a greater or lesser extent, producing a different kinematic combination [4, 5, 7, 15]. The resulting coordination pattern of the whole lower limb, therefore, might have to adapt in a non-conventional way to accomplish the motor task required for a given situation.

Other biomechanical variables also utilized to describe gait deviations in the diabetic population are less discriminant due to their controversial results [6]. While lower limb EMG and joint moments have been used to represent the causes of movement alteration and can potentially provide an understanding of how movement is affected, pattern definitions have not been developed. The findings in EMG and joint torques are still a matter of discussion, particularly when diabetic and polyneuropathic severity are being considered in the analysis [5, 15–21]. A more complex tridimensional model has also been developed to integrate kinetic and kinematic data, which may be claimed as a better tool to detect and describe gait changes in the diabetic population [22].

The majority of previous studies, however, have one major common characteristic: all have underrepresented the complexity of the studied phenomenon. While the locomotor task is a combination of non-linear interactions between the environment and disease responses through time, the usual biomechanical analyses reduce the complexity to a description of discrete and independent kinematic and kinetic variables; those variables may fail to adequately describe the entire gait cycle or its specific events. For instance, much of what is known about the gait patterns of diabetic patients comes from the analysis of peak values from ankle, knee, and hip kinematics, independent of each other, without considering the continuous nature of the task and the intersegmental interactions. The gait cycle is far more complex than can be described by a few discrete variables, especially ones represented by a global mean throughout the gait cycle or specific gait phases.

Deeper analysis of the quality of the cyclic system of gait generation could provide a better understanding of how mechanical changes are established and continuously maintained when diabetes has caused muscle dysfunction, joint restrictions, and reduction in sensory feedback. Combinations of these musculoskeletal and sensorial deficits in patients with different degrees of diabetic polyneuropathy may lead to a completely different kinematic matchup of the three main lower limb joints. In addition, distinct coordination patterns would be produced to overcome the mechanical challenges of walking with diabetic impairments.

Given these assertions, a dynamic systems approach has been used to investigate the non-linear characteristics of several cyclic biological phenomena, such as gait. For this particular approach, the continuous relative phase (CRP) technique is one of the most sensitive for detecting asymmetries in coordination during gait compared with other analyses of movement symmetry [23–26]. CRP has been effective in identifying cyclic movement deviations caused by several disabilities [27, 28] in different locomotor tasks [29, 30], but has seldom been utilized in the diabetic population.

The main goal of the study was to describe alterations in intralimb joint coordination patterns throughout the gait cycle that could distinguish between healthy individuals and different groups of patients with early, moderate, and severe stages of polyneuropathy. The study hypothesis were(i) different stages of severity would progressively result in more changes in the coordination pattern of the gait cycle, (ii) these alterations would be more evident in patients with more severe degree, and (iii) gait variability would be reduced for diabetic groups compared to healthy control groups. These findings may lead to the development of more effective therapeutics targeting the improvement of gait coordination and thus fewer falls and ulcerations.

## Methods

### Subjects

Sixty subjects between 45 and 65 years old were assessed and divided into 4 groups using a fuzzy logic model [31]: 13 healthy controls (CTRL, 55 ± 7 years, 1.66 ± 0.05 m, 66.5 ± 6.5 kg, 24 ± 2 kg/m^2^), 18 diabetic patients without neuropathy (DIAB, 59 ± 6 years, 1.65 ± 0.09 m, 77.2 ± 19.4 kg, 28 ± 6 kg/m^2^, 11 ± 7 years after diabetes diagnosis), 7 diabetic patients with mild neuropathy (MILD, 56 ± 4 years, 1.73 ± 0.06 m, 84.7 ± 9.7 kg, 27 ± 2 kg/m^2^, 19 ± 7 years after diabetes diagnosis), and 22 diabetic patients with moderate to severe diabetic neuropathy (SVRE, 57 ± 5 years, 1.66 ± 0.08 m, 80.0 ± 12.1 kg, 29 ± 4 kg/m^2^, 14 ± 8 years after diabetes diagnosis). The four studied groups did not differ in age (p = 0.392) or height (p = 0.183). Body mass differed among groups (p = 0.031): the CTRL group had lower body mass than the MILD (p = 0.0345) and SVRE (p = 0.017) groups. The body mass index also differ among groups (p = 0.021): the CTRL group presented lower body mass index than the MILD group (p = 0.123).

The criteria used for the diagnosis and staging of neuropathy were assessed based on the standardized questionnaire of the Michigan Neuropathy Screening Instrument [32]. The foot physical examination was visually performed to identify the presence of common signs and structural alterations that develop with DPN, such as callus, cracks, hallux valgus, hammertoes, claw toes, cavus or flat foot. Any history of foot ulceration or amputation was also registered. Tactile perception was assessed with Semmes-Weinstein monofilament of 5.07/10 g applied to a non-callused site under 4 areas of both feet (total of 8 foot areas): plantar surface of the distal phalanx of the hallux and the first, third, and fifth metatarsal heads. The examiner applied the stimulus arrhythmical and in a random order, the patient was asked to report the areas where the stimulus was felt [33]. Vibration perception was assessed by a timed method, according to Perkins et al [34], with a 128-Hz tuning fork applied bilaterally to the bony prominence of the interphalanx joint of the hallux. The patient reported when the perception of the vibration was not felt anymore. The examiner then noted the time (in seconds) that vibration sensation diminished beyond the examiner’s perception in the hand holding the tuning fork. Under 10 seconds was considered normal, and longer than 10 seconds was considered diminished. Perception was considered absent if the patient did not report feeling the vibration.

The fuzzy logic model classified diabetic patients based on the previously described clinical assessment, which included sensorial evaluation (tactile and vibration sensitivity) and typical symptoms of neuropathy into 4 categories of severity: (i) absence of neuropathy, (ii) mild neuropathy, (iii) moderate neuropathy, and (iv) severe neuropathy. For the present study, patients with no neuropathy were part of the DIAB group. Patients classified as mildly neuropathic were part of the MILD group, and individuals classified as moderately and severely neuropathic were grouped together in the SVRE group.

All participants gave their written, informed consent which was previously approved by the Ethics Committee of the School of Medicine of the University of São Paulo (Protocol number 054/10).

### Gait analysis protocol and data processing

Patients underwent biomechanical gait analysis while barefoot in a 10 m walkway. Passive-reflective markers were placed on the subject using a standard Cleveland Clinic marker set [35]. Extra markers for the static standing trial were placed bilaterally on the medial knee joint line, medial malleolus, and first metatarsal joint to determine relative joint centers of rotation for the knee, ankle, and longitudinal axis of the foot. These extra markers were removed in the dynamic gait trial. Three non-collinear reflective markers were also fixed at two squares, forming sets of technique clusters. One was placed in the lateral thigh and the other over the shank. The laboratory coordinate system was established at one corner of the force plate, and all initial calculations were based on this coordinate system. Based on the surface markers, each lower limb segment (foot, shank, and thigh), was modeled as a rigid body with a local coordinate system that coincided with anatomical axes. Translations and rotations of each segment were reported relative to neutral positions defined during the initial standing static trial.

The 3-dimensional displacements of the markers were evaluated with 6 infrared cameras (Optitrack FLEX: V100, Natural Point, OR, USA). Mathematical data analysis was performed using Arena (Natural Point, OR, USA) and Visual 3D software programs (C-Motion, Kingston, ON, Canada). Kinematic data were filtered using a fourth-order zero-lag low-pass Butterworth filter, with a cutoff frequency of 6.0 Hz.

All subjects were requested to maintain gait cadence between 96 and 120 steps/minute, but in a self-selected pace between trials. Statistical comparisons did not show significant differences in gait speed between groups (CTRL 1.16 ± 0.08 m/s, DIAB 1.12 ± 0.19 m/s, MILD 1.18 ± 0.14 m/s, SVRE 1.12 ± 0.08 m/s, p = 0.07). Data of one lower limb per subject, randomly chosen, were analyzed and compared. Five valid trials were acquired after total habituation of the subjects to the laboratory environment [36]. The means of the 5 trials were used for statistical purposes.

### Continuous relative phase

CRP was calculated for each gait cycle (stance and swing phases) [37]. Hip, knee, and ankle segmental angular velocities were calculated as the first derivative of the segment angular position. Angular position and velocity were normalized to 101 data points. These normalized position and angular velocity vectors were plotted relative to each other for each of the three joints. Phase angle for hip and ankle joints during the gait cycle were calculated using (θ_i = tan_^-1^[y_i_/x_i_]). Afterward, the relative phase angle was calculated by determining the difference between the distal and proximal joint phase angles (θ_relative phase = Φ distal segment– Φ proximal segment)_ [38].

CRP was defined for the following joint pairs: (a) ankle and hip, (b) ankle and knee, and (c) knee and hip. CRP variability was defined as the standard deviation of the CRP at each time point across the trials.

### Statistical analyses

For the CRP time series of the combined joints, one-way ANOVAs between groups were applied to each frame of the resulting time series based on Gaussian data distribution (Shapiro-Wilk test, p > 0.05), followed by Newman-Keuls post hoc tests and the Bonferroni correction, respectively.

For statistical purposes, the gait cycle was divided into five phases: early stance (0–10%), midstance (11–40%), propulsion (41–60%), initial swing (61–80%), and terminal swing (81–100%). ANOVAs between groups were applied to compare the standard deviation of the 5 gait phases, representing the CRP variability. Statistical analysis was performed with Statistica software (version 12, Statsoft Inc., Chicago, IL), with the significance level set at p < 0.05.

## Results

### CRP Analysis

**Fig 1:**
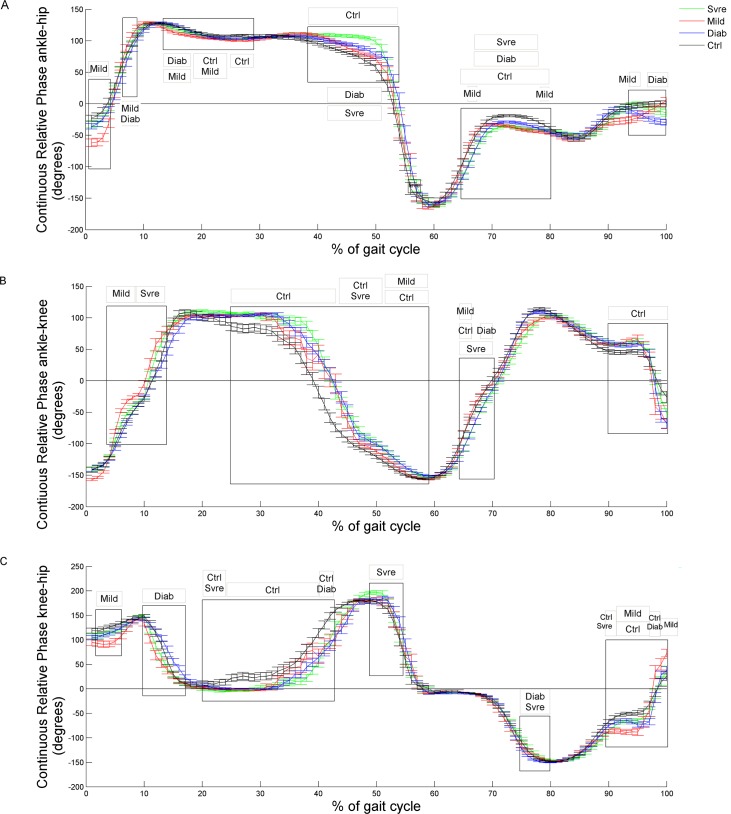
Mean and standard error of the Continuous Relative Phase for (A) ankle-hip, (B) ankle-knee, (C) knee-hip joint couples of CTRL (black), DIAB (blue), MILD (red), SVRE (green) groups. Frames of the time series that presented statistical differences are inside a rectangle (ANOVA). The small rectangles represent groups that differed from each other (post hoc analysis–Newman-Keuls). If 2 groups occupy the same small rectangle, they differed.

For the ankle-hip CRP (Fig 1A), the SVRE group presented an anti-phase ratio at the initial swing and propulsion phases which distinguished them from the DIAB and CTRL groups. At the midstance phase, the MILD group presented a different coordination pattern from the CTRL group. Qualitatively noteworthy at the early stance, propulsion, and terminal swing phases the coordination patterns of the diabetic groups differed considerably from each other and from the CTRL group, suggesting that neuropathic severity may influence ankle-hip kinematic adjustments.

For the ankle-knee CRP (Fig 1B), diabetic patients, with or without neuropathy and independent of its severity, presented a more equal anti-phase ratio than the CTRL group at the midstance, propulsion, and terminal swing phases. However, at the initial swing phase, all diabetic groups, particularly the neuropathic ones, presented in-phase coordination patterns. This is a very specific gait phase when the knee becomes the primary joint to perform distal limb clearance.

For the knee-hip CRP (Fig 1), the DIAB and MILD groups presented similar in-phase ratios throughout the gait cycle, except at the terminal swing phase.

### Variability analysis

**Fig 2:**
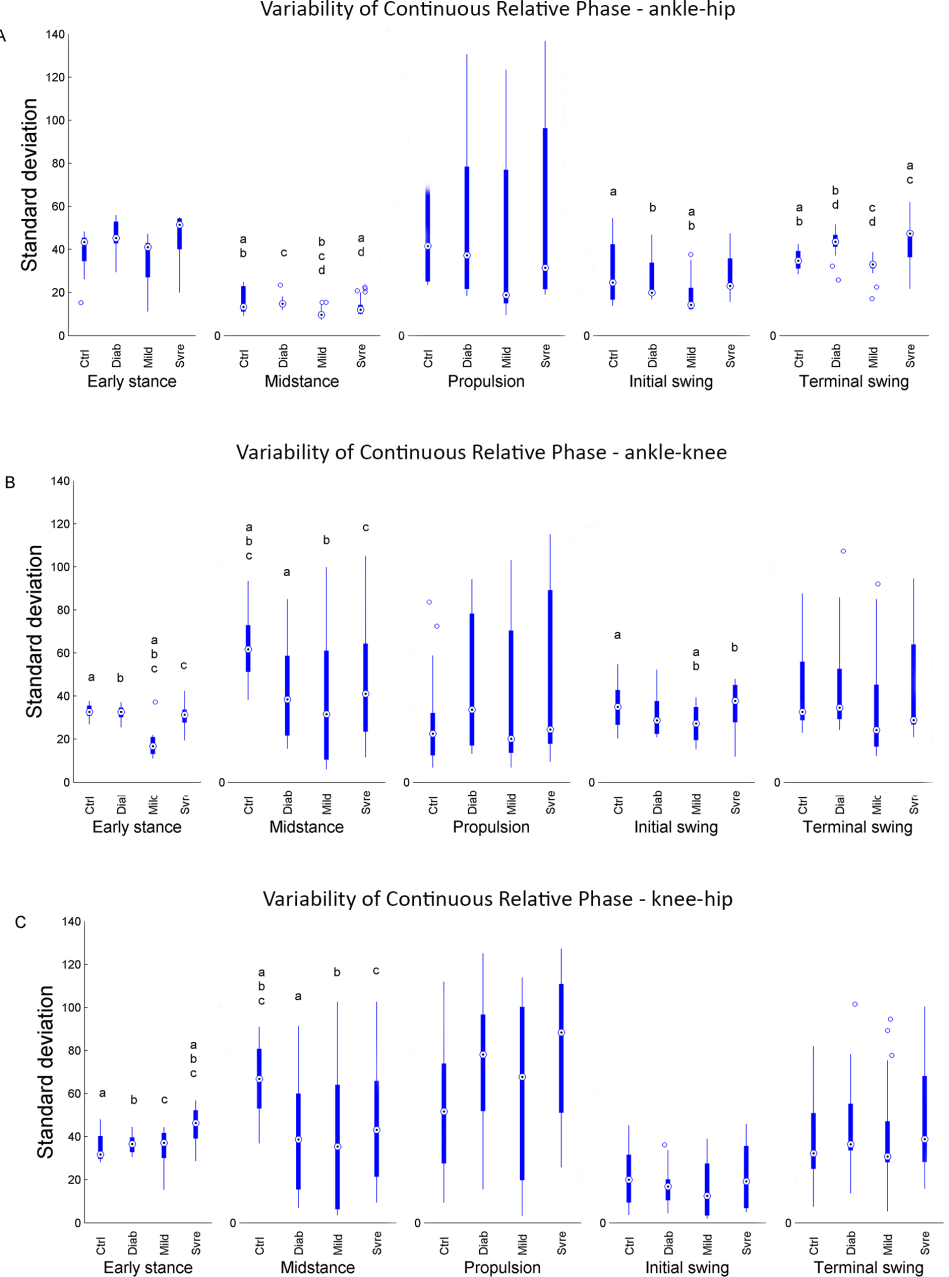
Box plots of CRP variability for (A) ankle-hip, (B) ankle-knee, and (C) knee-hip joint couples in the gait cycle, for CTRL, DIAB, MILD, and SVRE groups. The central mark is the median, the edges of the box are the 25th and 75th percentiles, the whiskers extend to the most extreme data points not considered outliers, and outliers are plotted individually. Differences between groups are represented by a couple of letters a, b, c, or d.

Results of the ankle-hip CRP variability (Fig 2A) showed that the MILD group presented lower variability than all other groups at the midstance, initial and terminal phases. The CTRL group showed higher variability compared to the SRVE group at the midstance and terminal swing phases. In the ankle-knee CRP variability data (Fig 2B), the MILD group also showed the lowest variability at the early stance phase, and showed lower variability at the initial swing phase compared to the CTRL and SVRE groups. During the midstance phase, the CTRL group presented the highest variability. At the early stance phase, the SRVE patients' knee-hip CRP variability (Fig 2C) was highest of all groups. At the midstance phase, the CTRL group presented the highest variability.

## Discussion

Diabetes Mellitus and polyneuropathy progressively lead to impairments in stability and joint motion during locomotor activities, and this study tested if those impairments also affect intralimb joint coordination patterns while walking. Differences in intralimb joint coordination among healthy controls, diabetic patients without neuropathy, diabetic patients with mild and diabetic patients with moderate to severe diabetic neuropathy were observed at the midstance phase in the ankle-hip CRP (when the limb has to provide the load transfer in unipedal support) and at the transition phase from stance to swing in both ankle-hip and knee-hip CRPs (when the hip plays an important role in toe clearance, and the knee assumes the key task of propelling the limb). Low variability and marked differences in CRP of all joint pairs in the MILD patients during the early stance phase were also observed, suggesting that gait coordination is altered not only when frank evidence of neuropathy is present, but also when neuropathy is still incipient. Particularly at the early stance phase, the ankle and tibialis anterior are crucial for controlling the flattening of the foot, a task already compromised in early phases of the disease [39]. Also notable, diabetic patients exhibited different ankle-knee CRPs than CTRL individuals even without the presence of neuropathy, suggesting that diabetes can influence lower limb coordination during gait.

In general, healthy subjects presented different CRP patterns than diabetic individuals from the midstance through terminal swing phases for both joint pairs that included the knee. Additionally, the same joint pairs at the midstance and propulsion phases presented lower variability for all the diabetic groups compared to the CTRL group, representing a distinct and less variable pattern in subjects with diabetes irrespective the presence of neuropathy.

The ankle-hip CRP pattern of SVRE patients at the propulsion and initial swing phases showed a more anti-phase ratio compared to diabetic patients without neuropathy and the CTRL groups. SVRE patients presented markedly different coordination patterns at the propulsion stage in ankle-hip, knee-hip, and ankle-knee CRPs. Watari et al. [31] showed that the worsening in tibialis anterior and vastus lateralis activation increases with disease progression, and in the late phase of neuropathy, the medial gastrocnemius presented a reduced magnitude and a delay in the activation peak, impairing the propulsion phase. SVRE group results might be explained by knee and ankle extensors impairment and the subsequent adjustments in the whole lower limb kinematics that SVRE patients showed in their coordination patterns.

While ankle-knee CRP distinguished the gait coordination pattern between healthy control patients and diabetic patients, it did not distinguish among the different stages of polyneuropathic severity. Ankle-knee CRP did, however, distinguish among different degrees of neuropathic severity at the initial swing phase, when the ankle finishes its role in propelling the body forward, and the knee becomes an important joint to perform the clearance of the distal part of the limb.

It was expected that the SVRE group's distal segments would be more compromised by the neuropathy, and therefore that the hip, a more preserved joint in the course of the disease, would have to deal with an insufficient functionality of the distal part of the lower limb. The greatest difference between the SVRE and other groups, however, was at the propulsion phase, when the hip is involved in both joint pairs, and the CRP remains in anti-phase longer than other phases. Longer anti-phase maintenance was also observed at the midstance and propulsion phases for ankle-knee CRP. This difference seemed to be a characteristic trait of the SVRE group, in addition to the main differences concentrated primarily at the end of the stance phase through the initial swing phase. Contrary to expectations, early stance phase coordination differences were not observed, despite prior descriptions of range of motion [4, 14, 40–46] and muscle activation changes [5, 17, 20, 21, 39, 42] for this population, especially at the ankle. At the early stance phase, the SVRE group presented significantly higher variability compared to the MILD group for both joint pairs that involve the knee, but this variability was similar to other groups without neuropathy.

Patients from the DIAB and MILD groups presented similar coordination patterns, mainly at the midstance phase, with intermediary variability compared to healthy and severely neuropathic patients. These two intermediate groups fall between completely healthy and more severely neuropathic, and might present a transition pattern of coordination during gait as they adapt to a new health condition (DIAB), search for joint angular strategies to deal with disease progression (MILD), and reconcile the mechanical demands of the gait task. This result suggests, as previously proposed [44], that knee kinematics and coordination patterns could be the main parameters that distinguish patients with intermediate stages of diabetes and neuropathy.

Significantly, MILD patients presented a distinct CRP pattern for all three joint pairs at the early stance phase, bolstering other authors' assertions, that distal impairments demand kinematic adjustments of the whole lower limb chain [46], particularly during ground contact and the ankle, knee, and hip need to eccentrically attenuate the loads. At early stance phase, the patient also has to deal with these loads and transfer them properly through the body to perform the task without collapsing [45]. Watari et al. [31] have also used EMG recordings to identify distal impairments in the gait phase of load acceptance where the tibialis anterior and vastus lateralis present reduced activation in diabetic patients without neuropathy. Therefore, the loss of sensitivity and proper muscle activation in MILD patients could affect the motion control of the whole kinematic lower limb chain and might be enough to differentiate this group from others.

In median and interquartile range movement variability, the gait phase with the greatest range of variation was clearly, when the lower limb contacted the ground during the midstance and propulsion phases, when weight-bearing demands require upward adjustments. This variability is necessary so that the somatosensory system can program strategies for joint positioning when touching the ground, allowing proper adjustment to the imposed disturbances when the lower limb is in a closed kinetic chain [45–48]. Interestingly, the variability among groups at this phase does not consistently trend up or down in line with disease severity, and did not demonstrate a well-defined progression of motion variability proportional to disease severity. Therefore, the hypothesis that movement variability would be influenced by the reduced range of motion and altered muscle control due to the neuropathy resulting in a restriction of the degrees of freedom was not confirmed. For patients classified as severe in the study, cases of amputation and ulcers in remission less than 6 months were excluded from analysis. These unanalyzed patients may demonstrate that the restricted ankle range of motion more clearly affects movement variability.

Moreover, the ankle-hip joint pair showed the lowest variability during the midstance phase. Even for the CTRL group, variability at this phase was markedly reduced when compared to the other two joint pairs, and was lower than the groups with diabetes and neuropathy. The midstance phase is characterized by a single support that should sustain and propel the body forward. This transfer occurs in the intermediary range of motion of the three major lower limb joints, which would be less affected by the movement amplitude restriction due to disease. Nevertheless, it is necessary to keep proper muscle activation to support the body weight in only one limb. It was expected, therefore, that there would be a greater variability secondary to the increased possibilities of joint movement combinations and to the greater muscular demand to perform this body transference. All groups, however, performed the movement with small variability; it may be described as a well-defined way of moving. The other joint pairs involving the knee showed increased variability, particularly in the diabetic groups, suggesting that the knee may have a greater influence on the movement coordination during the midstance phase than the hip and ankle.

Early kinematic modifications of gait in diabetic patients were observed in the metatarso phalangeal joints, as well as an impaired dynamic control with advancing of the diabetes [49]. Similar to Lamola's findings, in the presente study, the mild group showed low variability at the stance phase, in the CRP of the lower extremity, always involving the ankle: ankle-hip and ankle-knee CRPs, and this finding suggests that the diabetic neuropathy severity influences progressively kinematic adjustments in an ascending pattern—from the ankle to upper joints in the lower limbs.

A limitation of the current study is the small sample size. However in similar studies, using robust methodology, such as the recent of Lamola et al. (2015) [49], patients at different levels of involvement by diabetes mellitus, were distributed in their respective groups in similar numbers as we used in the present study. There were expected differences in body mass and body mass index between CTRL and mild and severe patients, because as the disease develops, the patients become heavier which is a risk factor for further complications in diabetes [50]. These differences might be a factor that could interfere in the kinematic pattern of the more severe patients; however, this is an anthropometric typical characteristic of those patients.

## Conclusion

The three CRPs were able to distinguish between the CTRL and diabetic groups at the midstance and propulsion phases, demonstrating that alterations in gait coordination are not only present when neuropathy is frankly evident, but also when neuropathy is incipient. Diabetes can change lower limb gait coordination patterns, especially in those relying on the hip joint. The ankle-hip coordination pattern distinguished SVRE patients from other diabetic patients, particularly in the transition phase from stance to swing when the limb has to deal with load transfer in unipedal support because the hip plays an important role in distal limb clearance. The ankle-knee coordination pattern differentiated between all degrees of neuropathic severity and the CTRL group at the initial swing phase when the knee assumes the key role in propelling the limb. Increased variability in ankle-knee coordination occurred at the gait phase when the foot contacted the ground and weight-bearing demanded upward adjustments. Although reductions in coordination variability in line with neuropathic severity were not observed as might be expected, the CTRL group did present decreased variability at the midstance phase compared to the diabetic groups. The variability in knee joint pair coordination became more distinct as neuropathy increased and the knee's role during gait changed as the disease progressed.
